# Admissions to Bushey Fields Hospital, Dudley

**DOI:** 10.1192/bjo.2025.10487

**Published:** 2025-06-20

**Authors:** Maryam Imran Aziz, Praveen Kumar

**Affiliations:** Bushey Fields Hospital, Dudley, United Kingdom

## Abstract

**Aims:** The on-call doctor at Bushey Fields Hospital, Dudley, has multiple responsibilities out-of-hours, including covering 5 wards (both general and older adults), clerking new admissions, attending reviews with the Psychiatric Liaison Team, receiving calls from CRHTT for medication advice, and carrying an arrest bleep for emergencies.

Resident doctors’ meetings have raised the issue of admissions frequently occurring out of hours, and that doctors are often unaware until the patient has arrived, giving less time to prepare. These lead to patients and ward staff waiting at late hours for assessment, delays in prescribing and administering medications, and increased workload for the on-call doctor.

The aims of this project were to: determine during which shifts patients are most commonly admitted; determine a timeline of when the bed is identified, when the patient arrived, and when clerking is completed; and look into how to involve doctors more in the process.

**Methods:** This was a retrospective audit conducted on admissions to all 5 wards at Bushey Fields Hospital from August to November 2024 (inclusive). The Trust audit department provided data, including patient demographics, admitting ward, day and time of admission, and whether this was in or out of hours. Out of hours shifts were established as any shift outside of 9 am–5 pm Monday to Friday.

Additional data was collected from notes on the Trust’s online system Rio; this included the date and time a bed was identified, when the clerking proforma was started, and what shifts these occurred on. 10 patients were selected at random from each ward, for a total sample size of 50 patients.

**Results:** During the time frame evaluated, there were 180 total admissions, of which 15% (27) were in hours, and 85% (153) were out of hours.

Of the 50 randomly selected patients, the bed was identified on a day shift for 50% (25), but 84% (42) were admitted out of hours. For 90% (45) of patients, the clerking process was started out of hours, and for 76% (38), the clerking document was started after the patient’s arrival.

**Conclusion:** As the results show admissions largely occur out of hours, we have suggested several recommendations. These include informing doctors of admissions beforehand, and for the patient’s parent team to pre-fill the clerking proforma sections about demographics, background and history to reduce the workload for the on-call doctor, as the document can be edited.

